# Nano-Antenna Coupled Infrared Detector Design

**DOI:** 10.3390/s18113714

**Published:** 2018-10-31

**Authors:** Mohamed H. Mubarak, Othman Sidek, Mohamed R. Abdel-Rahman, Mohd Tafir Mustaffa, Ahmad Shukri Mustapa Kamal, Saad M. Mukras

**Affiliations:** 1 School of Electrical and Electronic Engineering, Universiti Sains Malaysia, 14300 Pulau Pinang, Malaysia; moh.habashy@gmail.com (M.H.M.); othman.sidek@gmail.com (O.S.); 2 Electrical Engineering Department, King Saud University, Riyadh 11421, Saudi Arabia; mabdelrahman@ksu.edu.sa; 3 School of Physics, Universiti Sains Malaysia, 11800 Pulau Pinang, Malaysia; ashukri@usm.my; 4 Mechanical Engineering Department, Qassim University, Buraydah 51452, Saudi Arabia; mukras@qec.edu.sa

**Keywords:** antenna coupled detector, nano-antenna, bolometer, MOM diode

## Abstract

Since the 1940s, infrared (IR) detection and imaging at wavelengths in the two atmospheric windows of 3 to 5 and 8 to 14 μm has been extensively researched. Through several generations, these detectors have undergone considerable developments and have found use in various applications in different fields including military, space science, medicine and engineering. For the most recently proposed generation, these detectors are required to achieve high-speed detection with spectral and polarization selectivity while operating at room temperature. Antenna coupled IR detectors appear to be the most promising candidate to achieve these requirements and has received substantial attention from research in recent years. This paper sets out to present a review of the antenna coupled IR detector family, to explore the main concepts behind the detectors as well as outline their critical and challenging design considerations. In this context, the design of both elements, the antenna and the sensor, will be presented individually followed by the challenging techniques in the impedance matching between both elements. Some hands-on fabrication techniques will then be explored. Finally, a discussion on the coupled IR detector is presented with the aim of providing some useful insights into promising future work.

## 1. Introduction

Both mid-wave infrared (MWIR) and long-wave infrared (LWIR) bands, ranging between 3 to 5 and 8 to 14 μm respectively, are becoming increasingly important for various commercial and military applications. While these bands are strongly emitted by objects near 500 K and 300 K respectively, which include emission from humans and animals, as well as engines and machines [1], they also experience relatively low absorption in the atmospheric window [2]. These two reasons explain the great importance of these bands as well as the special interest in the design and development of detectors at these bands for different commercial and military imaging applications. While the longer band is generally attractive for its higher sensitivity, the latter is more attractive for higher contrast imaging purposes [3].

**Figure 1:**
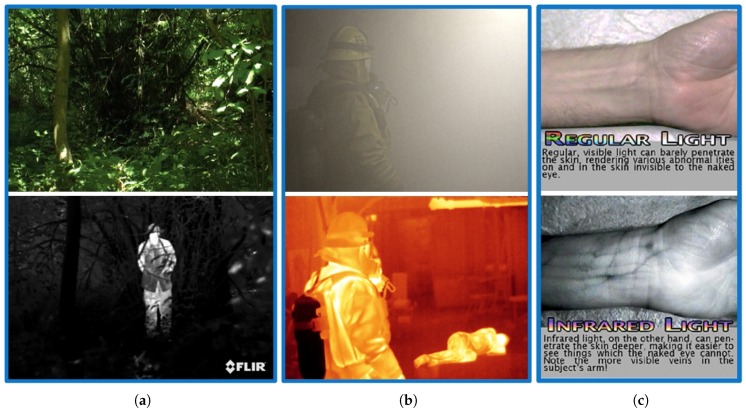
The effectiveness of using IR imaging compared to normal imaging in some real applications: (**a**) Surveillance [9], (**b**) Fire fighting [10], (**c**) Medical imaging [11].

Figure 1 catalogs the effectiveness of using IR imaging compared to normal imaging in some real applications such as surveillance, firefighting and medical imaging. Developments in the technology have led to its use in a variety of other applications such as mine detection, predictive maintenance, industrial process control and terahertz (THz) and infrared spectroscopy [1,4,5]. More recently IR detectors have also been proposed for energy harvesting applications, attracting the attention of many researchers [6,7,8].

To unlock the potential for more applications, detection without cooling was introduced in order to eliminate the requirement for bulky and expensive cryogenic cooling equipment. This facilitated the fabrication of compact and low cost detectors and thus enabling its use in more applications. Similarly, along with the uncooled detection, high-speed detection as well as spectral selectivity are now reported as the main required features for the new generation detectors. The development of the high-speed detectors paved the way for high frame rate cameras which offer a good solution for many critical applications such as target detection, tracking and navigation in autonomous vehicles. Moreover, with the spectral selectivity, dual-band cameras are achievable enabling clutter suppressed enhanced imaging applications such as missile warning systems to be more reliable [12]. Also, multi-spectral detection is beneficial for THz and IR spectroscopy application for chemical detection and materials characterization.

A review on nano-antenna coupled detectors, specifically as one of the state-of-the-art IR detectors, is presented in this work. To provide a solid background, the general consideration for designing the antenna coupled IR detectors will first be investigated. In this context, both antenna and the coupled localized sensing elements will be discussed individually. We will begin with a brief account of the nano-antenna and the challenging design issues in contrast to the classical planar RF antennas. Both metal-insulator-metal (MIM)diodes and microbolometers will be investigated as two examples of commonly used localized sensing elements. Matching techniques between the antenna and sensing element will then be described. The device fabrication process development will also be summarised. Finally, some expectations for possible future trends will be presented.

## 2. Antenna Coupled IR Detectors

**Figure 2:**
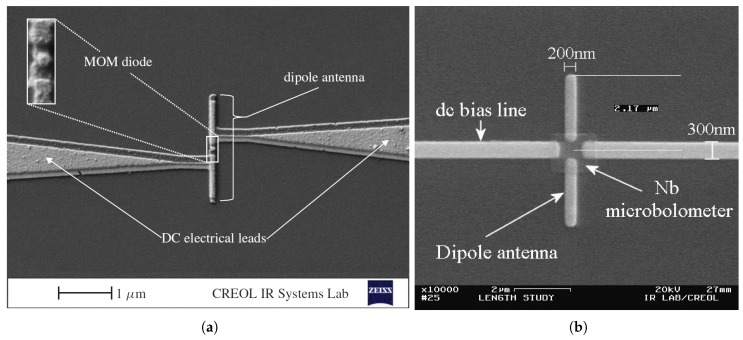
Scanning electron micrograph of a dipole antenna-coupled infrared detector example: (**a**) Antenna-coupled MOM diode [18], (**b**) Antenna-coupled bolometer [16].

Nano-antenna coupled detectors have been introduced as a new member in the IR detector family. The nano-antenna operating in the THz regime, is conceptually used to optimize the energy transfer from the freely propagating radiation field to a localized sensor which becomes much smaller than the operating wavelength [13,14]. The ultra-fast response as well as the ability to be operated without cooling, are both attributed to the submicron size of the sensor. Inherent advantages of nano-antenna include spectral selectivity as well as polarization sensitivity, that allow the elimination of heavy and bulky optical filters and polarizers (of magnitudes greater than 1 kg) which were previously required for commercial bolometric focal plane array IR cameras. Moreover, CMOS compatibility, extremely low design profile of these devices as well as the simplicity of the hardware circuit requirements, have made these detectors appealing and attracted the interest of researchers [1]. Antenna coupled IR detectors, such as antenna coupled bolometers [15,16,17], antenna coupled rectifiers [1,18,19,20,21], and antenna coupled thermocouples [22,23,24], have therefore received great attention from researchers. An example of the antenna coupled metal-oxide-metal (ACMOMD) as well as the antenna coupled bolometer detectors are shown in Figure 2.

Although many other competitors to these detectors have already been employed and can offer applicable solutions to meet the requirements for the new generation detectors, several difficulties that have limited the manufacturing yield of these detectors have been experienced. The critical epitaxial growth of mercury-based compounds required for the mercury cadmium telluride (MCT) detectors as well as air bridge and suspended structures required for the bolometric focal plane array are two examples of these difficulties in fabrication [25].

Due to the presence of the supporting substrate for the nano-antenna, both air-dielectric interface in addition to the new optical and electrical material parameters at the IR band result in performance degradation in antenna coupling efficiency and in turn, the whole device performance. Accordingly, power loss due to substrate modes, attenuation due to surface impedance and Coleman effects, as will be detailed later in Section 3.3 and Section 3.4, are all present [1,26]. This limited the specific detectivity D* of the antenna coupled detectors to values lower than $10^{7}\;\mathrm{cm}\;\cdot \;{\mathrm{Hz}}^{1/2}\;\cdot \;W^{-1}$ [27], which is at least about three orders of magnitude lower than its cryogenic-cooled IR counterparts [28]. The extremely low detectivity of these detectors is therefore the main impediment for these devices being widely commercialized. Considerable efforts had been made to improve the overall efficiency of the device either by increasing the antenna coupling efficiency [16,17,18,29,30,31], sensing device efficiency [18,20,32], impedance matching efficiency between both the antenna and the sensing device [22,33] or even optimizing the fabrication process [18,34].

## 3. Antenna Design

The nano-antenna will provide irradiance harvesting and optimize energy transfer to the sub-wavelength localized sensor. Antenna performance (gain, directivity, resonance, impedance, etc.) will then greatly influence the detector characteristics. The antenna shape and geometry can be tailored for given requirements including, for example, resonant wavelength, spectral response, polarization and angular response. Most of the challenges in the antenna design are encountered here mainly because of the air-dielectric interface. As a result, trapped surface waves will always be excited and thus reduce the radiation efficiency of the antenna and cause crosstalk between neighboring antennas [35]. Moreover, due to the new boundary conditions imposed on the electromagnetic (EM) fields through this interface, resonant wavelength, radiation pattern and antenna current distribution will all be changed as opposed to those placed in free space [20]. The electrical and optical properties of the materials will also be altered at the IR band representing another challenge in design. The finite effective electron mass in metals will result in delayed reaction to incident EM field as the frequency increases. The metals will cease to behave as perfect conductors. Although plasmon resonance cutoff frequency is observed sufficiently beyond the IR band, the conductivity of metals still drops considerably while skin depth increases at the LWIR band [13,36]. This will in turn result in attenuation, propagation losses and noise levels that have impact on antenna current distribution, especially for the antenna arrays whenever they are of design interest. As a result, the surface impedance of the metal strip and Coleman effect, which may also attribute the antenna current distribution and cause attenuation, has to be considered and cannot be neglected at this band. This has previously been neglected and excluded from the corresponding calculations [26].

In acquiring antenna design with dimensions that can fall in the range of tens of nanometers, the change of properties of the nanowire should also be considered. In such cases, as the dimensions may become comparable to the electron mean free path, electron scattering will increase and conversely thermal conductivity and thermoelectric properties of the metal will reduce [23,24]. The heat distribution over the structure can then influence the antenna temperature and the performance when coupled to thermal sensors such as bolometers or thermocouples.

### 3.1. Dielectric Half-Space and Angular Response

**Figure 3:**
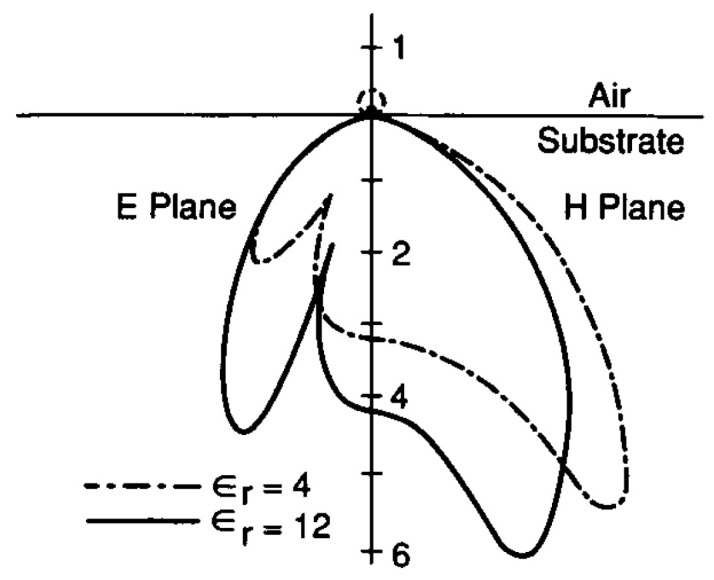
Radiation patterns of a resonant dipole on a semi-infinite dielectric substrate with $\epsilon_{r}$ = 4 and $\epsilon_{r}$ = 12 [37].

In contrast to the half wave dipole antenna operating in free space, the symmetry of angular response will be broken at the air-dielectric interface in the case of antenna coupled detectors. Therefore, as shown in Figure 3, the antenna angular response will show a higher sensitivity to the incident radiation from the half space with the higher dielectric function, ϵ [26,30] as follows:

(1) $$\frac{P_{sub}}{P_{air}}={\left(\frac{\epsilon_{sub}}{\epsilon_{air}}\right)}^{3/2}$$

where $P_{sub}$, $\epsilon_{sub}$, $P_{air}$, $\epsilon_{air}$ are power and permittivity in both substrate and air respectively. The gain and directivity of the designed antenna will also follow the same spatial division. It is fairly obvious that the substrate selection and the illumination configuration will then inherently influence the detector performance.

According to Equation (1), the higher radiation efficiency of the antenna will be towards the medium of higher permittivity. Accordingly, in order to achieve a higher gain it will then be advantageous to illuminate the receiving antenna from the substrate rather than the air-side [20]. A theoretical increase of 40 times in sensitivity is therefore expected in the case of a substrate-side illumination through the Si substrate. However, both illumination configurations have been studied experimentally and an average measured signal increase by a factor of 2.1 was only reported for substrate-side illumination [30].

Some problems may arise with the substrate-side illumination such as reflection losses, a narrower cone angle, and severe power loss due to generation of more substrate modes. Different substrate configurations have been engineered accordingly to mitigate such problems. For instance, the early designed architectures used a few hundred nanometer layer of silicon dioxide, SiO${}_{2}$, for thermal and electrical insulation [38]. The thickness of this layer was thereafter tuned for quarter wavelength matching [18,20,26,39]. In that way, the reflection was minimized and irradiance transmission of 79% was measured at 10.6μm through the Si substrate with 1.6μm SiO${}_{2}$ matching layer compared to only 48% without this layer. Benzo-cyclobutene (BCB) has also been used in place of SiO${}_{2}$ since its refractive index is close to that of air which enables air-side illumination configuration. BCB also has additional advantages including its relatively low loss at infrared wavelengths and ease of deposition via spin coating [29,40,41,42,43,44].

Ground plane and reflectors are proposed in various other configurations enabling air-side illumination. In this regard, a ground plane at the back of the substrate has been implemented to reflect back the radiation through the substrate to the device giving the benefit of higher sensitivity from the substrate-side [26].

On the other hand, according to image theory, the antenna image can result in an overall increase in response, higher antenna quality factor and narrower resonance [22,45]. To construct the antenna image, however, the antenna has to be patterned on top of a λ/4 thick standoff layer over the ground plane. Thus, the antenna image was addressed providing a promising compensation for the case of air-side illumination by many researchers [22,40,42,43,44].

Besides the influence of substrate configuration on the detector responsivity, its effects on the angular response for a single dipole has also been investigated and showed a wide range of full width at half maximum (FWHM) from 40° to 140° depending on the presented configurations [40].

**Figure 4:**
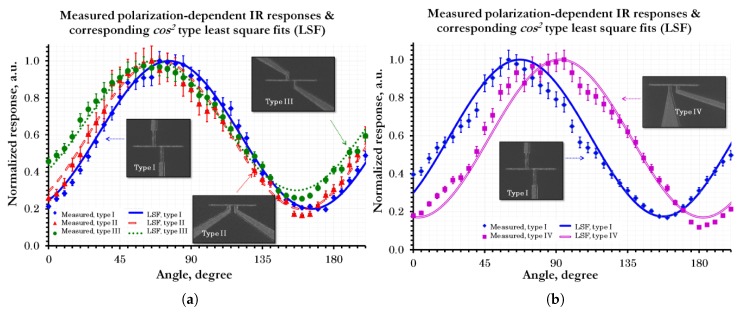
Polarization dependent measured responses of the ACMOMDs together with the corresponding cos${}^{2}$-type fits for different configurations of read-out interconnect designs: (**a**) Symmetrical configurations, (**b**) Asymmetrical configuration [46].

Along with the various forms of the substrate configurations, electrical leads may also contribute to the device performance radiation pattern as well as the polarization characteristics [46]. Due to their long size and large width compared to the antenna, however, they are believed to have no significant impact if proper electrical leads configuration is set together with a non-thermal sensitive element such as the MOM diode [47]. The influence of the leads on the polarization response of the ACMOMDs has been presented through different configurations of electrical leads as shown in Figure 4 [46]. On the other hand, the situation differs when the detector is coupled with thermal sensitive elements such as bolometer. The measured response will receive considerable contributions throughout the metallic structures connected to the bolometer unless the TCR of the bolometer is several orders of magnitude greater than those metals [43].

### 3.2. Antenna Effective Resonant Length

Due to the air-dielectric interface, the resonant length will be shifted from that expected in free space. At the dielectric interface, the wave tends to propagate with an intermediate velocity between those of the air and dielectric. This will result in an effective dielectric constant, $\epsilon_{eff}$, of

(2) $$\epsilon_{eff}=\frac{\epsilon_{sub}+\epsilon_{air}}{2}$$

Consequently, the effective wavelength $\lambda_{eff}$ is

(3) $$\lambda_{eff}=\frac{\lambda_{o}}{\sqrt{\epsilon_{eff}}}$$

where $\lambda_{o}$ is the radiation wavelength in free space. A resonant dipole of antenna length 3.1μm implemented on SiO${}_{2}$, for which the dielectric constant is 4.84, is expected to theoretically be the proper length that can show the first resonance to irradiance of wavelength 10.6μm [1]. However, since a semi-infinite substrate model is assumed in this theoretical approximation, the actual effective length will be shifted down due to actual finite substrate dimensions. According to simulation, actual resonant length in the order of 2.4μm has been published [23].

**Figure 5:**
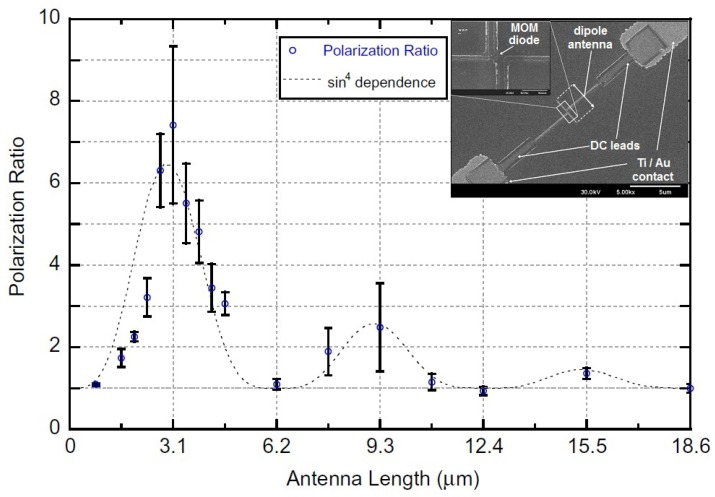
Polarization ratio of Al/AlOx/Pt ACMOMD as a function of the length of the dipole antenna [1].

The higher order resonance has also been observed but with exponentially attenuated amplitudes. The exponential attenuation beyond the first resonance is attributed to surface impedance and the Coleman effect that influence current distribution as will be detailed later. The antenna normalized response given in terms of polarization ratio, which is defined as the ratio of the maximum (co-polarized) to the minimum (cross-polarized) response, has been fitted to a theoretical attenuated response as a function of antenna length, $L_{dip}$, (see Figure 5) with a total resultant attenuation constant, $\Gamma_{exp}$, expressed as follows [1,30]:

(4) $$Response\propto \sin^{4}\left(\frac{\pi L_{dip}}{2\lambda_{eff}}\right)\exp\left(-\frac{\Gamma_{exp}}{2}L_{dip}\right)$$

**Figure 6:**
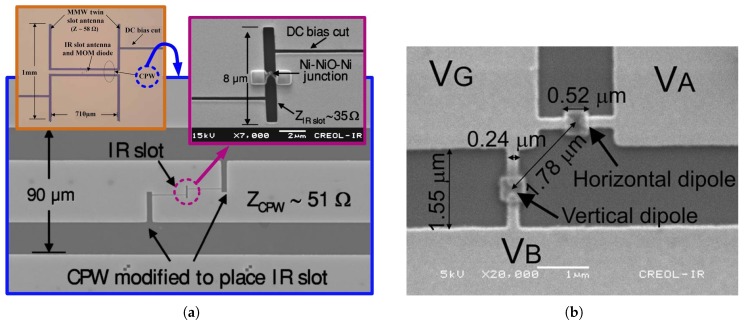
Dual antenna structures: (**a**) MMW/IR dual band antenna [48], (**b**) Orthogonal dipole antenna for elimination of cross polarization response [15].

Along with the single resonant wavelength antenna, different dual feeding structures have been developed for dual band imaging at both IR and millimeter-wave (MMW) frequencies. For instance, IR slot and MMW twin slot antennas have been integrated through a coplanar waveguide (CPW) as shown in Figure 6a [48]. The measured $D^{*}$ of $1\times 10^{6}\;\mathrm{cm}\;\cdot \;{\mathrm{Hz}}^{1/2}\;\cdot \;W^{-1}$ at the IR band has been reported for a similar dual band structure of IR dipole and MMW slot antennas [21,49]. Another dual feeding structure of orthogonal dipole antenna, as shown in Figure 6b, has also been implemented and incorporated as two elements in a Wheatstone bridge to mitigate the cross polarization response [15].

**Figure 7:**
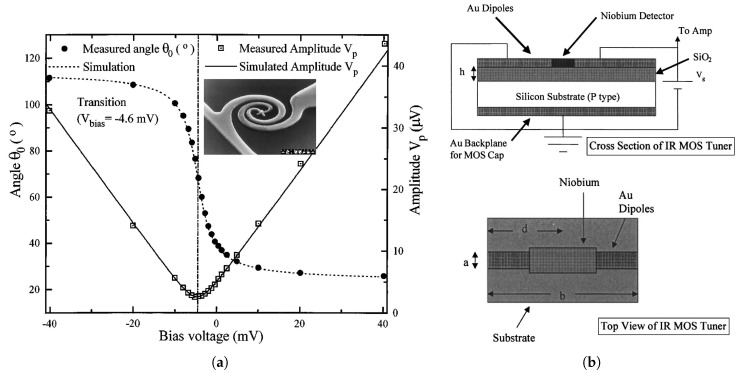
Tunable antenna coupled IR detectors: (**a**) Polarization tunable measured response with different bias voltages [50], (**b**) Illustration of MOS tuner structure [52]

Moreover, while the spectral, polarization and angular responses are defined and limited by the antenna shape and geometry, various solutions have been proposed to enable controllability of different antenna characteristics. In this context, polarization tunable detectors have previously been proposed and implemented achieving a $90^{\circ }$ polarization tuning of a spiral antenna (Figure 7a) with the aid of a ±40mV tuning voltage, that controls the capacitance of the coupled contact diode [50,51]. Similarly, wavelength tunable detectors have also been demonstrated and showed a tuning range of 0.5μm near 10μm with the aid of a 300mV tuning voltage, $V_{g}$ (see Figure 7b), that controls the MOS capacitance underneath the dipole antenna arms [52].

### 3.3. Surface Waves

**Figure 8:**
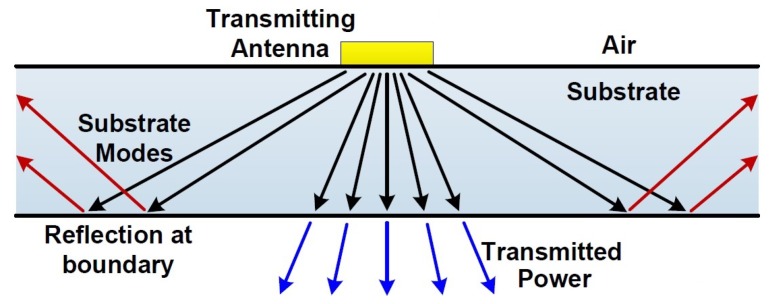
Ray illustration of trapped surface waves excitation in a substrate.

Surface waves, also known as substrate modes, are generated as excited trapped waves due to total reflection of waves that are incident beyond the critical angle [53,54]. Figure 8 gives a brief explanation for the generation of surface waves for a transmitting antenna lying on a substrate according to the ray point of view. As these waves propagate transversally and scatter at bends and surface discontinuities [55], they result in performance degradation as well as cross talk between neighboring antennas. Usually, a lower radiation pattern due to the resultant power loss and a radiation pattern distortion due to multiple lobes and spikes are expected together with a lower polarization ratio [55].

The number of allowable guided modes as surface waves and, consequently, the associated losses can be minimized by increasing the cutoff frequency, $f_{c}$, of the lowest order transverse electric (TE) mode which is given by [54]:

(5) $$f_{c}=\frac{c}{4d_{sub}\sqrt{\epsilon_{sub}-1}}$$

where *c* and $d_{sub}$ are the velocity of light in free-space, and thickness of the antenna substrate respectively. The inverse proportionality to the substrate thickness suggests that substrates thinner than 1μm are desirable for operation at 10.6μm. Accordingly, this requirement will add some difficulties to the fabrication process [26].

To reduce the degradation due to the substrate modes, the coherence of the reflected waves at the Si-air interface has been broken up by roughening the back side of the planar substrate [56]. This technique limits only the propagation of the surface waves, however, the power loss will still exist.

Attaching a lens behind the substrate results in a surface wave loss reduction as it allows the transmitted waves to experience a small incidence angle on the lens surface [35]. Accordingly, the hemispherical lens substrate has been used with substrate side illumination [17]. A low-loss material and quarter-wave matching layer on the lens surfaces should be applied to minimize the expected losses. A substantial increase in detectivity has been reported for ACMOMD illuminated from the substrate side through Ge lens which showed $D^{*}$ of $9.65\times 10^{6}\;\mathrm{cm}\;\cdot \;{\mathrm{Hz}}^{1/2}\;\cdot \;W^{-1}$ in this case compared to $1.91\times 10^{5}\;\mathrm{cm}\;\cdot \;{\mathrm{Hz}}^{1/2}\;\cdot \;W^{-1}$ for the case of air-side illumination [18].

**Figure 9:**
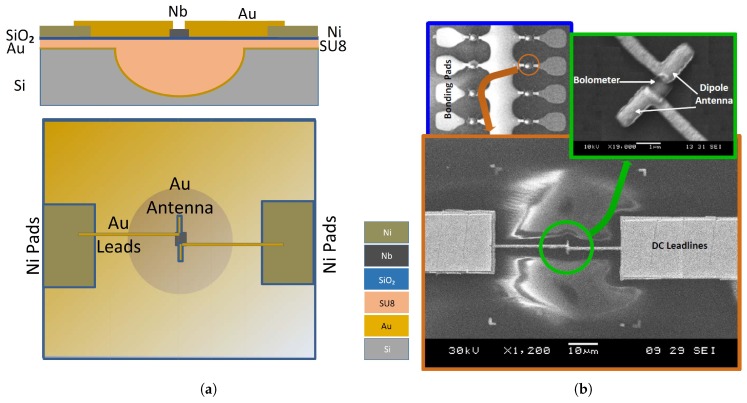
Parabolic reflector cavity backed structure: (**a**) Demonstrative diagram (**b**) Scanning electron micrograph (SEM) of a complete integrated device [57].

Another technique that can substantially reduce surface wave degradation is by using either a circular or rectangular shaped integrating cavity, at the back of the substrate [55]. In this context, the parabolic reflector integrated structure has been proposed as illustrated in Figure 9 [57]. Besides reducing the substrate mode losses, that structure also offers high polarization discrimination response and increased effective antenna aperture area that adds a space diversity gain to the detector and inherently increases its detectivity [58]. A preliminary simulation analysis of a similar structure has shown a 20 dB gain increase compared to the conventional structure [59].

### 3.4. Antenna Current Distribution

Various levels of antenna current degradation are encountered in the IR band. As mentioned earlier, antenna current distribution will be influenced by surface resistance and the Coleman effect. While the former is due to the skin effect on metal strips, the latter is the current exponential attenuation as a result of phase velocity changes at the air-dielectric interface through its propagation along the antenna length [47]. The attenuation constant for surface resistance $\Gamma_{sr}$ is given by:

(6) $$\Gamma_{sr}=\frac{2\pi }{\lambda_{o}}\kappa$$

where κ is the imaginary part of the complex index of refraction of the antenna metal. On the other hand, the Coleman effect attenuation constant $\Gamma_{c}$ is given by:

(7) $$\Gamma_{c}=\frac{2\pi }{\lambda_{o}}\sqrt{\frac{\epsilon_{sub}+\epsilon_{air}}{2}}$$

Moreover, the current will also be resolved into longitudinal and transverse components influenced by the width of the metal strips that forms the nano-antenna. Due to the non-circular cross section of the antenna arms, a transverse current component will be generated together with the main longitudinal component [60]. To reduce the transverse currents and maintain only longitudinal antenna currents, the width $w_{dip}$ of the metal strips should be very small compared to the length of the antenna or free-space wavelength in other words. According to the analysis presented by [60], the aspect ratio, $\frac{w_{dip}}{L_{dip}}$, of 0.2 will reduce the transverse current to two orders of magnitude lower than the longitudinal component. Also according to the simulation results reported by [26], a width of 35 times smaller than the free-space wavelength or even narrower, is expected to result in negligible transverse currents, and hence a better antenna performance. The dipole antenna width should, therefore, be less than 330 nm for a desired wavelength of 10.6μm [61].

### 3.5. Antenna Arrays and Effective Aperture

Since the antenna geometrical design is constrained to the wavelength of interest, the size of the antenna is required to be of the order of a few micrometers. The effective antenna aperture, which is of the order of $\lambda^{2}$ [62], will then represent the effective irradiance area of the detector. Low achievable normalized detectivity stems from the lack of collection efficiency due to that small effective area, which is measured in the case of a 3.1μm half-wavelength dipole to be around $61\;\mu m^{2}$ [47].

**Figure 10:**
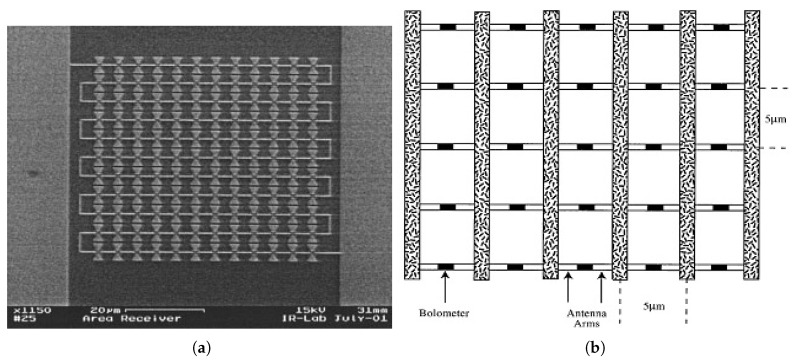
Effective collection area enhancement approaches: (**a**) Antenna array in serial configuration [39], (**b**) Antenna array in parallel configuration [63].

Various solution approaches have been proposed to overcome the challenge of attaining a larger effective area while maintaining the sub micron size of the sensing element. Different array configurations of antenna coupled detectors have been implemented in serial, parallel, or even two dimensional hybrid scheme representing a single pixel detector as shown in Figure 10 [39,63,64]. These approaches are, however, of limited interest as the noise level is expected to build up due to the increased number of sensing elements.

**Figure 11:**
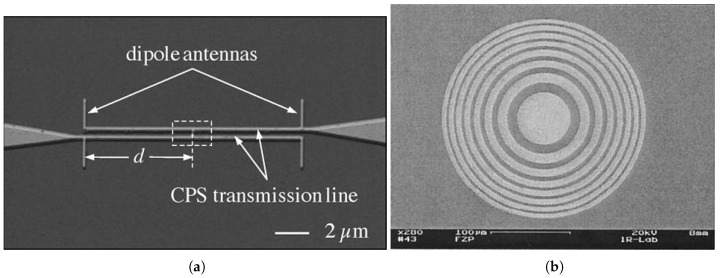
Effective area increase approaches: (**a**) Two elements phased array [44], (**b**) Fresnel zone structure [62].

In contrast to the aforementioned area receiver array of antennas, the phased array antenna, as shown in Figure 11a, employs multiple coherent antenna elements for space diversity as well as tailoring the angular response of the detector. Only a single sensing element is coupled to the array through CPS offering better matching and avoiding the increased level of noise. However, the limitation on propagation length due to high losses through metal wires at the LWIR band suggests that both antenna and sensor needs to be as close as possible and thus limits the size of the array. Another approach is to increase the effective aperture of the antenna individually. This has been proposed by integrating Fresnel zone plate lens (FZPL), as shown in Figure 11b, in the backside of the substrate to the antenna coupled detector that is implemented on the top of the substrate. The circular and square FZPL, were both implemented as integrated to spiral antenna coupled bolometer [62].

**Figure 12:**
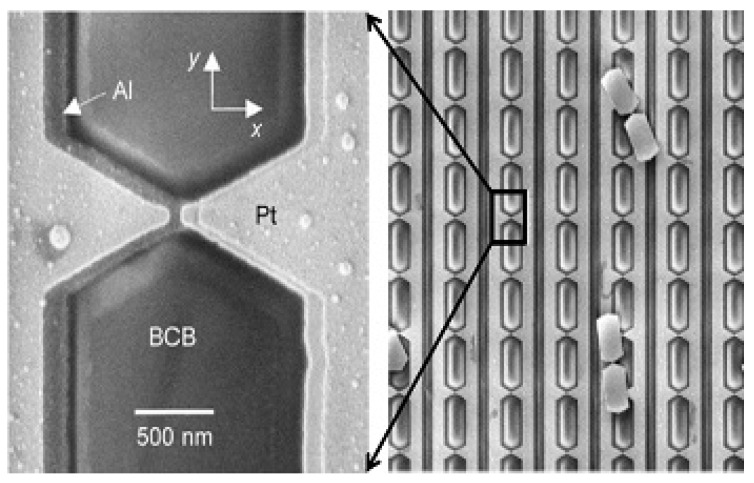
Frequency selective surface using slot antenna coupled to MOM diode [65].

Frequency selective surface in the form of slot antenna coupled to MOM diode, as shown in Figure 12, is another technique that has been employed to increase the effective area and maintain the fast time response from one point of view while simultaneously increasing the response through tailoring the transmittance, reflectance and absorbance [65].

## 4. IR Sensing Element

**Figure 13:**
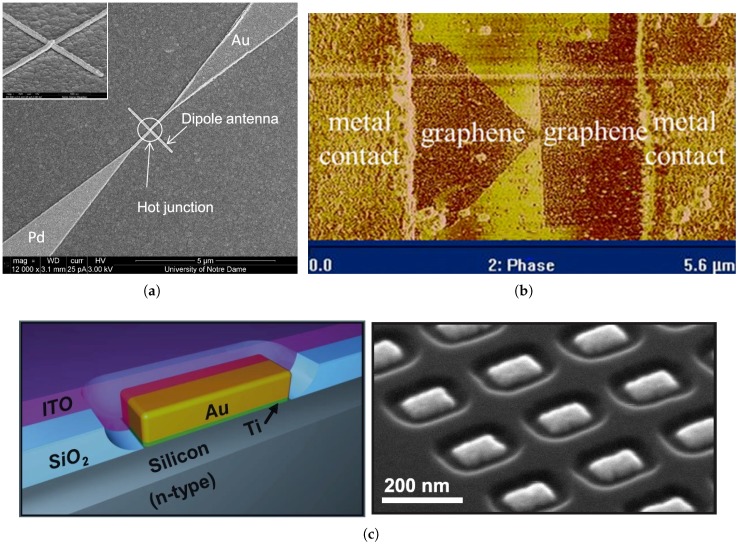
Examples of some different sensing techniques: (**a**) Thermocouple [23], (**b**) Geometric diode [68], (**c**) Schottky diode representation (left) and fabricated device before ITO coating (right) [70].

Many devices have been employed for IR detection with the antenna coupled integration. The quantum tunneling current rectification, bolometric response and thermoelectric response are the most commonly used techniques to sense the resonant current collected by the antenna. The novel ballistic rectification regime in graphene has also been employed recently in the LWIR band with its great potential to substantially reduce the device response time [66]. Examples of these devices include, but are not limited to, MOM diodes in a wide range of configurations, microbolometers, thermocouples [23,24], geometric diodes [66,67,68], Schottky diodes [69,70], quantum dot photodetectors [71,72,73] and others. Some of these devices are illustrated in Figure 13.

In this section, the MOM diode and microbolometer will be reviewed as two example models for the sensing elements highlighting some different design issues that are generally encountered for sensor design. For either element, the size becomes very small compared to the wavelength, and consequently a fast response and low-noise room-temperature operation are possible.

### 4.1. Metal Oxide Metal (MOM) Diode

**Figure 14:**
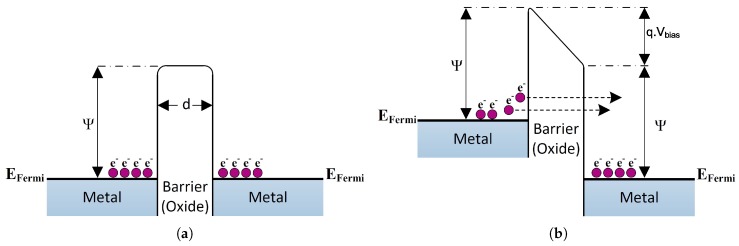
Energy band diagram for MOM contact: (**a**) Barrier formation, (**b**) Trapezoidal shaped barrier due to biasing effect.

MOM diodes, also known as metal-insulator-metal (MIM) or metal-barrier-metal (MBM) diodes [74], are implemented by simply sandwiching a thin oxide layer between two metals forming a potential barrier. For extremely high barrier and ultra-thin oxide barrier, conduction current arises due to the dominating quantum mechanical response and consequently non-linear I-V characteristics are observed. Figure 14a shows the potential barrier formed by the oxide layer as well as the biasing effect. Different configurations for the junction’s metals, either symmetric or asymmetric, have previously been studied. Some examples, found in the literature, of configurations that have been implemented include: Ni/NiO/W, Ni/NiO/Pt [74], Ni/NiO/Ni [75], Ni/NiO/Au, Cr/CrO/Au, Ni/NiO/Cu [76], Cu/CuO/Cu [77], $\mathrm{Al}/{\mathrm{AlO}}_{x}/\mathrm{Ti}$, $\mathrm{Al}/{\mathrm{AlO}}_{x}/\mathrm{Ni}$ and $\mathrm{Al}/{\mathrm{AlO}}_{x}/\mathrm{Al}$. Although external bias is required to generate an electric field inside the barrier, in the case of asymmetric junction, a built in electric field will be generated due to the electrodes’ work function difference without any externally applied bias. In either case, as shown in Figure 14b, a trapezoidal shaped potential barrier between the electrodes will be formed promoting the electrons above the Fermi level in the raised electrode to have a higher probability of tunneling. As a result, there will be a net electron flow through the junction [19].

When IR or optical radiation is coupled to a biased MOM, an additional alternating time dependent voltage is induced creating a perturbation to the field inside the barrier. This perturbation will modulate the Fermi levels in both metals, one with respect to the other to enhance the tunneling probability. Since this mechanism is an inherently fast process, it is believed to be responsible for rectification in the IR band. MOM diodes are thus capable of rectifying high frequency signals in the LWIR, MWIR and even up to the optical range [18].

The MOM devices show nonlinear I-V characteristics that are best described by Simon’s model for the low and intermediate voltage range [78]. The DC rectified current, $I_{r}$, can be derived as the time average of the current when expanded in a Taylor power series with respect to voltage [44]. It can hence be evaluated as:

(8) $$I_{r}={\left.\frac{1}{4}\frac{d^{2}I}{dV^{2}}\right|}_{V=V_{b}}{V_{0}}^{2}$$

where $V_{b}$ is the bias voltage and $V_{0}$ is the amplitude of the induced voltage. It is thus clear that the rectified current is proportional to the square of the amplitude of the optical voltage, and therefore, to the power of the incident infrared radiation at the MOM junction [43]. The proportionality constant is the second derivative of the I-V characteristic, which represents the non-linearity, evaluated at the bias voltage. Since the diode resistance, $R_{D}\left(V\right)$, is defined as the first derivative of the I-V characteristic, the rectified response can be rewritten in terms of the rectified voltage as follows:

(9) $$V_{r}={\left.\frac{1}{4}\frac{d^{2}I}{dV^{2}}/\frac{dI}{dV}\right|}_{V=V_{b}}{V_{0}}^{2}$$

The ratio between the second to the first derivative of the I-V characteristic, which is the proportionality constant in this case, is defined as the sensitivity, $S_{v}$, or the curvature of the diode.

According to Simon’s model, zero bias tunneling resistance, $R_{t}\left(0\right)$, is exponentially dependent on the barrier thickness as follows [20,79]:

(10) $$R_{t}\left(0\right)\propto \sqrt{\frac{s^{2}}{\bar{\phi }}}\exp\left(\sqrt{s^{2}\bar{\phi }}\right)$$

where *s* is the barrier thickness and $\bar{\phi }$ is the height of the rectangular barrier.

Moreover, the metal’s spreading resistance also adds more contribution to the zero bias resistance, such that, the total zero bias resistance of the diode, $R_{D}\left(0\right)$, is assumed to be the sum of the tunneling resistance and spreading resistance of the electrode’s metal. This can be approximated and expressed as follows:

(11) $$R_{D}\left(0\right)=R_{t}\left(0\right)+R_{s}=\frac{\sigma_{t}}{\pi a^{2}}+\frac{\rho }{2a}$$

where $R_{s},\;a,\;\sigma_{t}$ and ρ are the spreading resistance, contact area, tunneling resistivity and metal conductivity respectively [20,49]. The tunneling resistivity is thus a function of the barrier height, dielectric thickness and relative permittivity. It is clear that the zero bias resistance is inversely proportional to the contact area. Both the fabrication process and the metal selection will thus play an important role on controlling the resistance value. Since the contact area is bounded from above by the cutoff frequency, increased diode resistance is expected while lowering the contact area acquiring a faster diode response. This will in turn impose greater difficulty in the impedance matching between the diode and the antenna. Moreover, the sensitivity of the MOM diode is also inversely proportional to the diode resistance according to Equation (9). It is for this reason, that the diode resistance has to be efficiently lowered.

**Figure 15:**
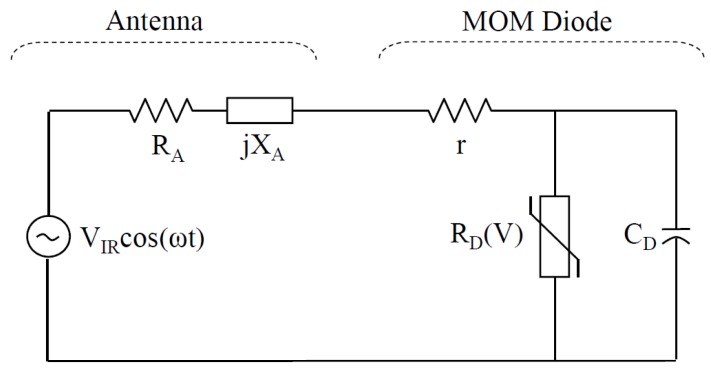
Equivalent-circuit model of an antenna-coupled MOM diode.

In its general form, as shown in Figure 15, the MOM equivalent circuit can be simply represented by a resistor-capacitor shunt circuit, with an extra series resistance, r, that represents leads. Cutoff frequency for antenna coupled MOM diode is thus limited by the diode capacitance such that the cutoff frequency, $f_{cut}$, is given by:

(12) $$f_{cut}=\frac{R_{A}+r+R_{D}\left(V\right)}{2\pi \left(R_{A}+r\right)R_{D}\left(V\right)C_{D}}$$

where $R_{A}$ and $C_{D}$ are the real antenna impedance part and the diode shunt capacitance respectively.

For the thin-film MOM diode, the junction’s capacitance is modeled as a small classical parallel-plate capacitor which can be expressed by:

(13) $$C_{D}=\frac{\epsilon_{ox}a}{d_{ox}}$$

where $\epsilon_{ox}$ and $d_{ox}$ represent the electrical permittivity and the thickness of the oxide layer, respectively. To increase the cutoff frequency, the device capacitance should be minimized. Since the oxide thickness is constrained with tunneling requirements, minimizing the device area is the only possible strategy to reduce the device capacitance. A typical area in the order of $0.01\;\mu m^{2}$ resulting in capacitance of 0.1 fF will thus be convenient for tunneling at 30 THz [20].

Breakdown voltage is another critical issue that arises due to the ultra-thin dielectric thickness. In the Al/AlOx/Pt asymmetric diode, for example, a built-in field of approximately 6.8MV/cm across $2\;\mathrm{nm}\;{\mathrm{AlO}}_{x}$ will be generated [18]. As the oxide and its barrier can be damaged if the field across the oxide exceeds the maximum dielectric strength, these devices are very sensitive to static charge accumulation, low frequency pickup voltages and transients in the circuit [80]. However, the breakdown voltage of the oxide depends greatly on the quality of the deposition method. An example for breakdown voltage enhancement from 1.4MV/cm to over 500MV/cm for a $5\;\overset{˚}{A}$ thin oxide layer depending on the oxide formation method has been reported [61].

Although the rectification in the MOM contacts was referred back to the dominant tunneling current, it had been claimed recently that the thermocouple effect is the most dominant [81]. However, this claim needs further investigation and verification especially since many different junctions have been measured and showed good agreement with the theoretical model including symmetrical junctions which is not included in that claim.

### 4.2. Bolometers

The second type of sensors in this review, which is also commonly used, is the bolometer. The word bolometer is derived from the word ’bolē’ which means ray meter in Greek [4]. It was introduced for the first time in 1889 by Samuel P. Langley for solar measurements. It is a type of thermistor that is developed for IR band detection such that its electrical resistance measurement change indicates the amount of temperature change due to the incident IR. On the other hand, in the case of antenna coupled bolometer, the change in resistance indicates the change in temperature as a result of Joule dissipation of antenna arm THz induced current that flows in the bolometer [82].

According to the analysis of conventional bolometers, the responsivity of the bolometer is mainly dependent on its material as well as its size. In this context, to produce large electrical resistance change by the absorbed radiation, the material of the bolometer is required to have a large temperature coefficient of resistance (TCR) and a very small thermal conductance, *G*, which is defined as the reciprocal of the change in temperature for each unit change in the total power dissipated in the bolometer. While the TCR is an intensive property that is dependent only on the bolometer’s material, the thermal conductance is an extensive property that is also proportional to the size of the device as well as the mounting technique. Decreasing the area of the bolometer’s cross section and increasing the thermal link’s length will result in increased thermal resistance and in turn increased responsivity [83,84].

TCR is normally positive for metals and negative for semiconductors. Several materials have previously been used for IR detection, including for example but not limited to, amorphous silicon (a: Si), silicon germanium (SiGe), silicon germanium oxide (SiGeO), vanadium oxide $\left({\mathrm{VO}}_{x}\right)$, yttrium barium copper oxide (YBaCuO), and some different metals [85]. Some TCR values for some of these materials are listed in Table 1. Although the TCR of bulk semiconductors is shown to be much higher than their metal counterparts, the electrical to thermal conductivity ratio for semiconductors is 2 to 4 orders of magnitude lower than that of metals. To increase this ratio, whose product with TCR is a more precise indication of bolometer detectivity, some semiconductor doping can be tailored in order to yield a result comparable to that of metals [86].

The output voltage responsivity was derived by solving the heat flow equation as given in detail in [53]. The bolometeric voltage responsivity is thus given by [27]:

(14) $${ℜ}_{v}=\frac{i_{bias}R\alpha_{b}\eta_{b}}{G\sqrt{1+\omega^{2}\tau^{2}}}$$

where $i_{bias}$ is the bolometer’s bias current, *R* is the bolometer’s resistance, $\alpha_{b}$ is the material’s TCR, $\eta_{b}$ is power absorption efficiency, and ω is the incident radiation’s modulation frequency. τ is the thermal time constant and is given by:

(15) $$\tau =\frac{C}{G}$$

where *C* is the heat capacity, or thermal mass, which is defined as the amount of heat added to the bolometer that will result in a temperature change of unity.

In general, to lower the background noise, a very small amount of the bias current is needed to prevent any additional heat that may be generated by the bias current. Therefore, the temperature coefficient or the thermal conductance are the only two parameters that can be tailored in the bolometer to increase the responsivity. While the first one is achievable by proper selection of bolometer’s material, its effect is significantly low and shows only a small percentage increase, compared to the effect of the latter parameter which can vary over several orders of magnitude. Hence, tailoring the thermal conductivity, by proper isolation as well as proper size selection, is expected to have higher impact on device responsivity despite having a trade off with the response time [53].

Analogous to the conventional bolometer, the antenna coupled bolometer is also subject to the same finding from the above analysis. While the absorption efficiency of the conventional bolometer is attributed to the optical property of the bolometer, it can be considered as the total antenna efficiency in antenna coupled bolometer detection. In terms of the other parameters, high TCR value and device resistance are similarly required while maintaining the thermal conductance and the DC bias current as low as possible. Between metals, niobium is more frequently used as a bolometer for operation at room temperature in the IR band due to its large TCR (0.005 K${}^{-1}$) as well as its high electrical resistivity of $1.5\times 10^{-7}\;\Omega m$ [45]. Though of little use, other metals have also been used as microbolometers coupled with antenna, such as the use of nickel in the LWIR band [15,39], as well as titanium [88] and bismuth [89] which have been used in the THz and far-infrared bands respectively.

**Figure 16:**
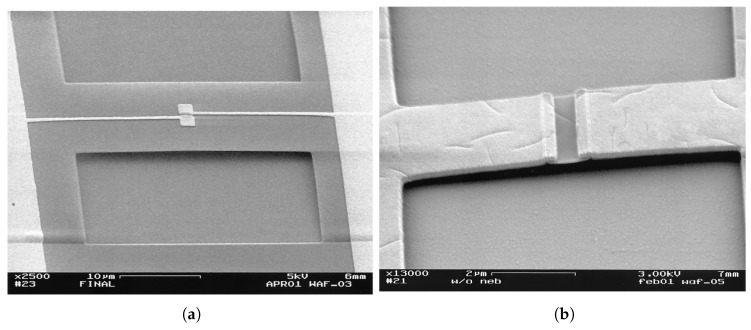
Air-bridge for isolation [45]: (**a**) Bolometer and antenna suspended on the air, (**b**) Suspended bolometer only.

In the search for a better thermal isolation technique, an air-bridge was implemented for some detectors to mitigate absorption in the insulating layer [45,86]. Examples of such detectors are shown in Figure 16. By implementing Ni bolometric spiral antenna on a ${\mathrm{Si}}_{3}N_{4}$ membrane as an air bridge, ref [27] attained a $D^{*}$ of $2.89\times 10^{7}\;\mathrm{cm}\;\cdot \;{\mathrm{Hz}}^{1/2}\;\cdot \;W^{-1}$ compared to $5.69\times 10^{5}\;\mathrm{cm}\;\cdot \;{\mathrm{Hz}}^{1/2}\;\cdot \;W^{-1}$ without the membrane.

Another isolation technique involved the use of silica gel. Compared to SiO${}_{2}$, the aerogel silica of 85% porosity can add one order of magnitude in detectivity for antenna coupled bolometer [84].

## 5. Impedance Matching Consideration

**Figure 17:**
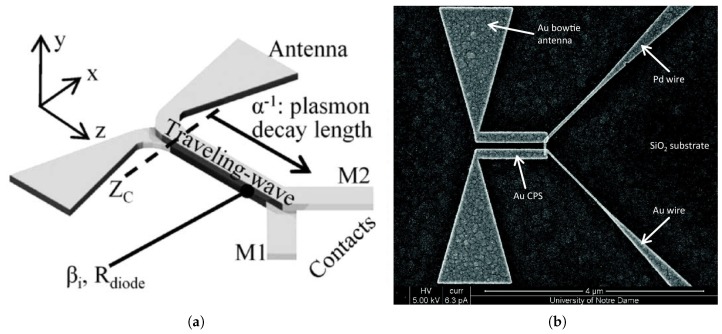
Impedance matching examples: (**a**) Traveling wave distributed MOM structure [33], (**b**) CPS to match antenna with thermocouple [22].

To efficiently couple the antenna harvested energy to the load, and achieve maximum power transfer, both antenna and sensing device have to be designed with matched impedance. Considering the antenna-coupled MOM device, which is represented by the equivalent circuit shown in Figure 15, two emerging contradictory considerations arise. Referring to Section 4.1, while a smaller contact area is required to reduce device capacitance for enhancing the device bandwidth, the diode resistance will dramatically increase as a result of the submicron size contact area. To circumvent this tradeoff, implementation of MOM diode with oxide having low resistance-area product such as NiO was initially proposed. This resulted in a product in the order of $1\;\Omega \;\cdot \;\mu m^{2}$ and consequently achieved good matching to 15–150 Ω as the typical antenna total resistance range. Nickel experiences high losses at IR band making it a poor preference for antenna [90]. Another proposed matching technique is to use travelling wave detector structure that employs the technique of rectifying a surface-plasmon wave, as shown in Figure 17a [33]. Yet, these aforementioned techniques are constrained only to high impedance devices. Another different matching strategy, as shown in Figure 17b, is to couple the device to antenna through a coplanar strip, CPS, which acts as a transmission line. This technique provides greater design flexibility covering a wider range of impedances that accommodates the ultra low impedance thermocouple as well as the MOM diodes of hundreds of kilo ohms. Such an approach has been studied and applied to various designs [22,29,39,91].

According to the length of CPS, *l*, the load impedance $Z_{L}$ will be transferred to the antenna as a new input impedance, $Z_{in}\left(l\right)$, as follows [39]:

(16) $$Z_{in}\left(l\right)=Z_{o}\frac{Z_{L}\cosh\left(\gamma l\right)+Z_{o}\sinh\left(\gamma l\right)}{Z_{o}\cosh\left(\gamma l\right)+Z_{L}\sinh\left(\gamma l\right)}$$

where $Z_{o}$ is the characteristic impedance and γ is the complex propagation constant. The characteristic impedance and complex propagation constant can be calculated as a function of the CPS design geometry as given in the literature [92,93]. It is thus obvious that the new input impedance at the antenna side can be matched by simply changing the length of the CPS as well as its characteristic impedance and complex propagation constant [22,54]. Tuning the CPS length for better matching resulted in a measured 2.4 fold increase in the response of the antenna coupled thermopile, while tailoring CPS characteristic impedance is simulated and yet expected to add an extra 7.2 times increase in response [22].

## 6. Device Fabrication

While reliable and reproducible high resolution structuring was the major concern for researchers, other different considerations, including high volume production with increased reliability, reducing the implementation complexity, or even enhancing the overall performance, were also addressed through process development. With the continuous advancement in nano-lithographic technology, different fabrication techniques have been employed. These include electron beam lithography (EBL), focused ion beam (FIB) milling, nano-imprint lithography (NIL) [34], and colloidal lithography [94].

Although EBL is the most commonly used lithographic technique, it often involves a lift-off process, which can be restrictive in creating nanostructures with high aspect ratios [95]. To ease the lift-off process, a bilayer e-beam resist, that is composed of a high contrast resist on top of higher sensitivity resist, is frequently used to introduce an undercut below a sufficiently thick bilayer resist. This will provide a clean separation of the deposited metals on top of the resist and in the voids, and will result in higher pattern resolution as well as cleaner edged patterns. As a rule of thumb the thickness of the resist should be at least three times the thickness of the deposited film to get the best results using lift-off [84].

Polymethyl methacrylate (PMMA) and methyl methacrylate-methacrylic acid (MMA-MAA) are the most common resist pair used for bilayer structure. Nevertheless, other resist pairs such as polydimethyl glutarimide (PMGI) and ZEP 520A-7 can also be used for the same purpose as reported by [27]. It is fairly obvious that the optimal exposure dose will have a very significant impact on achieving the required undercut profile as the behavior of PMMA may change from a positive to a negative tone if overexposed to more than 10 times the optimal dose [84].

Implementing both antenna and sensor element separately requires high resolution multi-layer lithographic processes with a complicated alignment process. This requirement presents a significant challenge for the device fabrication. To overcome this challenge, a fabrication process was developed to facilitate the implementation of these detectors through a single EBL process. The process involves patterning the ACMOMD by applying the shadow mask technique [32]. Along with offering a single EBL process, this technique also enables implementation of MOM diode with smaller contact. In this technique, the higher speed of the copolymer is traded for the higher resolution of PMMA resulting in a larger undercut in the copolymer that diffuses underneath the PMMA to form free-standing bridges of PMMA. Applying a normal incidence directional deposition twice, each with a slight wafer tilting in an opposite angle to each other, results in the formation of a very small contact area under the bridge [96]. In addition to offering an easier fabrication process, this technique also enables the formation of the oxide layer of MOM diode under the same vacuum before introducing the second metal layer deposition [18]. This, therefore, provides a highly controlled oxide thickness of high quality.

**Figure 18:**
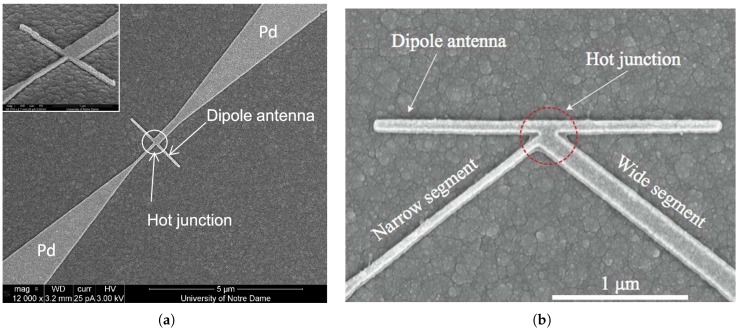
Examples of single metal antenna coupled nano-thermocouple [23,24].

While the effort in developing a single EBL process is convenient for the MOM diode, it may not be applicable for other devices such as bolometer coupled ones. Moreover, with the development of other device structures, this consideration may be totally resolved. For instance, as shown in Figure 18, by engineering only discontinuities and geometrical designs with dimensions below or even close to the electron mean free path, Seebeck coefficient can be tailored enabling single metal thermocouple structure [23,24,81].

## 7. Conclusions

Antenna coupled IR detectors are increasingly researched as promising candidates for the fourth generation IR detectors. The main design considerations for these detectors have been investigated throughout this review with regards to the antenna design, sensor design and their impedance matching. The fabrication challenge and development have also been highlighted. Since the research in this field is still in its infancy, a lot of research effort is still required to develop the technology.

A majority of the approaches addressed for the development of these devices have been reviewed in this work and summarized in Table 2. To unlock the potential of these detectors, the main emphasis of researchers have been to enhance detectivity to a level where they become comparable or exceed their commercially available counterparts. There are, however, other possible approaches that have the potential to dictate future research trends in this field. Developing electronically controlled devices, such as beam steering or wavelength tuning capabilities, is an example of the directions that may need to be researched more extensively.

According to the reciprocity theorem, nano-antenna can be regarded in its transmitting as well as receiving mode and, therefore, it can also be employed for antenna coupled emission. This concept has already been applied in the visible range [97,98,99,100], but it has not yet been deeply researched in the IR band. Antenna coupled IR emitters have a great potential to replace the bulky radiation source in applications, such as spectroscopy, promoting a more compact and portable solution.

Future research trends with regards to detector applications are also expected to emerge particularly when detectors become more commercialized as well as more affordable. One such example is low-grade thermal waste energy harvesting. This is anticipated to become a promising research area considering the analogy of solar energy harvesting. Although solar cells have been well established for energy harvesting which offers clean alternatives for fossil fuels, IR energy harvesting is perhaps more attractive since it can work during the daytime and nighttime. The authors speculate that integrating the single metal nano thermopile with the parabolic reflector will dramatically contribute towards the development of such harvesters.

The IR detection applications in medical imaging have been studied as it offers non-contact, non-invasive, ultra-fast, painless and safe imaging. It has been extensively researched for a wide range of applications starting from simple fever monitoring, blood pressure monitoring, diabetes mellitus, tumor and malignancy detection, including applications in dentistry, dermatology, orthopedics, rheumatology to gynecology, as well as ophthalmology, and even thermoencephaloscopy for functional imaging of the brain [104,105,106,107]. However, the presented studies are limited to two-dimensional imaging only. Three-dimensional imaging may require higher detectivity and more controllability in terms of spectral, polarization and angular responses. In this way, these different parameters can be employed for addressing the whole space of each layer in the image. Therefore, antenna coupled detection may have a significant impact that makes it likely to surge further in enhancing medical imaging as an alternative to computed tomography CT and magnetic resonance imaging MRI.

More applications are expected to emerge leading to new research fronts. It is the hope of the authors that this review will contribute to this field through informing the interested researcher about the state of the art with regard to detectors as well as attract and guide researches new to this field.

## Figures and Tables

**Table 1:** Temperature coefficient of resistance of some materials [87].

Material	TCR (K${}^{-1}$)
Vanadium Oxide	−0.02 to −0.03
Semiconducting YBCO	−0.0229 to −0.0337
Y-Ba-Cu-O (YBCO)	0.5 to 1
Ag	0.0037
Ni	0.005
Au	0.0036
Bi	−0.003

**Table 2:** Summary of different designs presented in the literature and their contributions.

Contribution/Study	Structure & λ(μm)	Measurement
Fabricate lithographic antenna, 1991 [38]	Spiral & Nb bolometer @ 9.5μm	$NEP=7\times 10^{-11}\;$ W
Develop the matching layer, 1994 [26]	Dipole & Ni MOM @ 10.8μm	transmission increase 31%
Fabricate on air membrane, 1997 [86]	Log-periodic & poly-Si @ 10.2μm	Directivity = 9 dB
Polarization tunable device, 1998 [50]	Spiral & MOM @ 10.6μm	Polarization response
Substrate-side illumination, 2000 [101]	Dipole & Nb bolometer @ 9.2–10.8μm	${ℜ}_{v}$ increase
Wavelength tunable device, 2001 [51]	Microstrip & bolometer @ 10.6μm	Spectral response
Fabricate on silica gel (Isolation), 2003 [84]	Bowtie & Nb bolometer @ 10.6μm	${ℜ}_{v}$ increase 20%
Develop MMW/LWIR dual band device 2004 [21]	Dipole & MOM @ 10.6μm	$D^{*}=1\times 10^{6}\;$ cm Hz${}^{1/2}$ W${}^{-1}$
Fabricate on $Si_{3}N_{4}$ bridge (Isolation), 2006 [27]	Square spiral & Ni bolometer & Ni bolometer @ 9–11μm	$D^{*}=2.89\times 10^{7}\;$ cm Hz${}^{1/2}$ W${}^{-1}$
Fabricate on hemispherical lens (Surface waves), 2006 [102]	Dipole & V bolometer @ 10.6μm	${ℜ}_{v}$ increase 11.7×
Fabrication using a single EBL (Process optimization), 2008 [32]	Dipole & MOM @ 10.6μm	$D^{*}=2.15\times 10^{6}\;$ cm Hz${}^{1/2}$ W${}^{-1}$
Develop traveling wave diode (Impedance matching), 2010 [33]	Traveling wave MIM TWMIM @ 3μm	$D^{*}=4\times 10^{6}\;$ cm Hz${}^{1/2}$ W${}^{-1}$
Study substrate configurations, 2010 [40]	Dipole & MOM @ 10.6μm	Radiation pattern
Develop beam steerable device, 2010 [43]	Phased array & MOM @ 10.6μm	Radiation pattern
Optimize MOM barrier formation (Process optimization), 2011 [18]	Dipole & Al/Pt MOM @ 10.6μm	$D^{*}=9.65\times 10^{6}\;$ cm Hz${}^{1/2}$ W${}^{-1}$
Develop CPS coupled device (Impedance matching), 2012 [22]	Bowtie & thermocouple with CPS @ 10.6μm	$D^{*}$ increase 2.4×
Simulate Seebeck antenna, 2014 [103]	Square spiral & thermocouple @ 10.6μm	Efficiency simulation
Develop a rectenna using graphene geometric diode (Response time), 2014 [66]	Dipole & geometric diode @ 10.6μm	$D^{*}=2.6\times 10^{6}\;$ cm Hz${}^{1/2}$ W${}^{-1}$
Develop a single metal/EBL device, 2015 [24]	Dipole & thermocouple @ 10.6μm	Spectral response
Fabricate parabolic reflector antenna(Surface waves), 2018 [57]	Parabolic reflector & Nb bolometer @ 10.6μm	Fabrication process recipe
